# 2-Ethyl-5-nitro­aniline

**DOI:** 10.1107/S1600536809009878

**Published:** 2009-03-25

**Authors:** Yan Chen, Zheng Fang, Ping Wei

**Affiliations:** a College of Life Science and Pharmaceutical Engineering, Nanjing University of Technology, Xinmofan Road No. 5 Nanjing, Nanjing 210009, People’s Republic of China

## Abstract

The mol­ecule of the title compound, C_8_H_10_N_2_O_2_, is nearly planar [maximum deviation of 0.163 (3) Å for one of the O atoms of the NO_2_ group]. In the crystal structure, weak inter­molecular N—H⋯N and C—H⋯O hydrogen bonds link the mol­ecules into chains, forming *R*_2_^2^(10) ring motifs.

## Related literature

For a related structure, see: Corwin (1955 ▶). For bond-length data, see: Allen *et al.* (1987 ▶). For ring motifs, see: Bernstein *et al.* (1995 ▶).
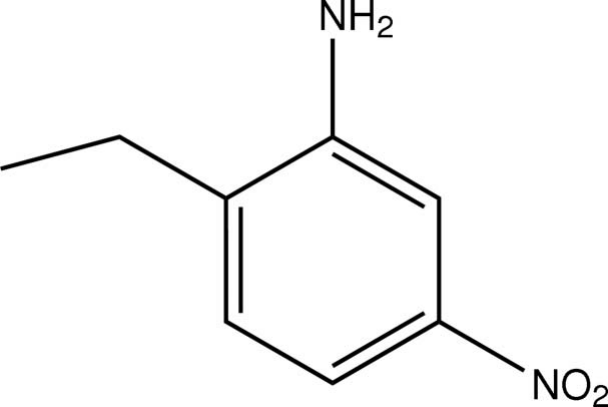


## Experimental

### 

#### Crystal data

C_8_H_10_N_2_O_2_
*M* *_r_* = 166.18
Monoclinic, 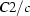

*a* = 23.037 (5) Å
*b* = 3.9540 (8) Å
*c* = 18.393 (4) Å
β = 104.51 (3)°
*V* = 1621.9 (6) Å^3^
*Z* = 8
Mo *K*α radiation
μ = 0.10 mm^−1^
*T* = 298 K
0.20 × 0.10 × 0.10 mm

#### Data collection

Enraf–Nonius CAD-4 diffractometer
Absorption correction: ψ scan (North *et al.*, 1968 ▶) *T*_min_ = 0.980, *T*_max_ = 0.990
2937 measured reflections
1474 independent reflections
906 reflections with *I* > 2σ(*I*)
*R*_int_ = 0.056
3 standard reflections frequency: 120 min intensity decay: 1%

#### Refinement

*R*[*F* ^2^ > 2σ(*F* ^2^)] = 0.055
*wR*(*F* ^2^) = 0.153
*S* = 1.01
1474 reflections
109 parameters
H-atom parameters constrained
Δρ_max_ = 0.23 e Å^−3^
Δρ_min_ = −0.21 e Å^−3^

### 

Data collection: *CAD-4 Software* (Enraf–Nonius, 1989 ▶); cell refinement: *CAD-4 Software*; data reduction: *XCAD4* (Harms & Wocadlo, 1995 ▶); program(s) used to solve structure: *SHELXS97* (Sheldrick, 2008 ▶); program(s) used to refine structure: *SHELXL97* (Sheldrick, 2008 ▶); molecular graphics: *ORTEP-3 for Windows* (Farrugia, 1997 ▶) and *PLATON* (Spek, 2009 ▶); software used to prepare material for publication: *SHELXTL* (Sheldrick, 2008 ▶).

## Supplementary Material

Crystal structure: contains datablocks global, I. DOI: 10.1107/S1600536809009878/hk2644sup1.cif

Structure factors: contains datablocks I. DOI: 10.1107/S1600536809009878/hk2644Isup2.hkl

Additional supplementary materials:  crystallographic information; 3D view; checkCIF report

## Figures and Tables

**Table 1:** Hydrogen-bond geometry (Å, °)

*D*—H⋯*A*	*D*—H	H⋯*A*	*D*⋯*A*	*D*—H⋯*A*
N2—H2*B*⋯N2^i^	0.86	2.62	3.423 (3)	156
C7—H7*A*⋯O1^ii^	0.93	2.60	3.417 (4)	147

Symmetry codes: (i) 
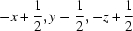
; (ii) 
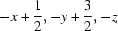
.

**Fig. 1.**
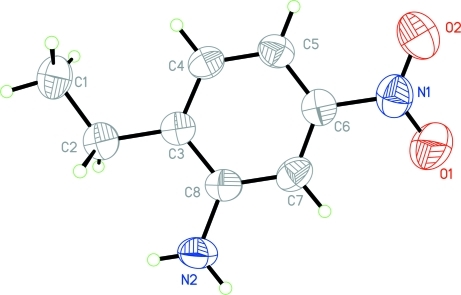
The molecular structure of the title molecule, with the atom-numbering scheme. Displacement ellipsoids are drawn at the 30% probability level.

**Fig. 2.**
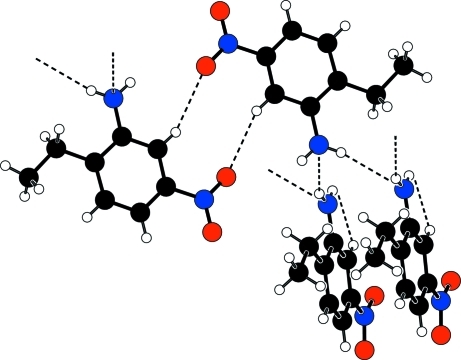
A partial packing diagram of the title compound. Hydrogen bonds are shown as dashed lines.

## supplementary crystallographic information

### Comment

Some derivatives of aniline are important chemical materials. We report herein the crystal structure of the title compound.

In the molecule of the title compound (Fig. 1), the bond lengths (Allen *et al.*,  1987) and angles are within normal ranges. Ring A (C3-C8) is, of course, planar. Atoms C1, C2, N1, N2, O1 and O2 are -0.067 (3), -0.028 (2), -0.035 (3), -0.055 (3), 0.054 (3) and -0.163 (3) Å away from the ring plane of A, respectively.

In the crystal structure, weak intermolecular N-H···N and C-H···O hydrogen bonds (Table 1) link the molecules into chains, forming R_2_^2^(10) ring motifs (Fig. 2) (Bernstein *et al.*,  1995). in which they may be effective in the stabilization of the structure.

### Experimental

For the preparation of the title compound, 2-ethylaniline (12.1 g) was dissolved in concentrated sulfuric acid (50 ml). The mixture was cooled to 273 K, and nitric acid (6.37 ml) was added in small portions. The mixture was stirred at 295 K for 0.5 h. Then, it was poured into a large volume of ice, used natrium hydroxydatum to neutralize excess acid, filtered, and dried. The compound was crystallized from cyclohexane (Corwin, 1955). Crystals suitable for X-ray analysis were obtained by slow evaporation of a methanol solution.

### Refinement

H atoms were positioned geometrically, with N-H = 0.86 Å (for NH_2_) and C-H = 0.93, 0.97 and 0.96 Å for aromatic, methylene and methyl H, respectively, and constrained to ride on their parent atoms, with U_iso_(H) = xU_eq_(C,N), where x = 1.5 for methyl H and x = 1.2 for all other H atoms.

### Crystal data

**Table:**

C_8_H_10_N_2_O_2_	*F*(000) = 704
*M**_r_* = 166.18	*D*_x_ = 1.361 Mg m^−^^3^
Monoclinic, *C*2/*c*	Mo *K*α radiation, λ = 0.71073 Å
Hall symbol: -C 2yc	Cell parameters from 25 reflections
*a* = 23.037 (5) Å	θ = 10–13°
*b* = 3.9540 (8) Å	µ = 0.10 mm^−^^1^
*c* = 18.393 (4) Å	*T* = 298 K
β = 104.51 (3)°	Block, colorless
*V* = 1621.9 (6) Å^3^	0.20 × 0.10 × 0.10 mm
*Z* = 8	

### Data collection

**Table:**

Enraf–Nonius CAD-4 diffractometer	906 reflections with *I* > 2σ(*I*)
Radiation source: fine-focus sealed tube	*R*_int_ = 0.056
graphite	θ_max_ = 25.3°, θ_min_ = 1.8°
ω/2θ scans	*h* = −27→27
Absorption correction: ψ scan (North *et al.*, 1968)	*k* = 0→4
*T*_min_ = 0.980, *T*_max_ = 0.990	*l* = −22→22
2937 measured reflections	3 standard reflections every 120 min
1474 independent reflections	intensity decay: 1%

### Refinement

**Table:**

Refinement on *F*^2^	Primary atom site location: structure-invariant direct methods
Least-squares matrix: full	Secondary atom site location: difference Fourier map
*R*[*F*^2^ > 2σ(*F*^2^)] = 0.055	Hydrogen site location: inferred from neighbouring sites
*wR*(*F*^2^) = 0.153	H-atom parameters constrained
*S* = 1.01	*w* = 1/[σ^2^(*F*_o_^2^) + (0.06*P*)^2^ + *P*] where *P* = (*F*_o_^2^ + 2*F*_c_^2^)/3
1474 reflections	(Δ/σ)_max_ < 0.001
109 parameters	Δρ_max_ = 0.23 e Å^−^^3^
0 restraints	Δρ_min_ = −0.21 e Å^−^^3^

### Special details

**Table:**

Geometry. All e.s.d.'s (except the e.s.d. in the dihedral angle between two l.s. planes) are estimated using the full covariance matrix. The cell e.s.d.'s are taken into account individually in the estimation of e.s.d.'s in distances, angles and torsion angles; correlations between e.s.d.'s in cell parameters are only used when they are defined by crystal symmetry. An approximate (isotropic) treatment of cell e.s.d.'s is used for estimating e.s.d.'s involving l.s. planes.
Refinement. Refinement of *F*^2^ against ALL reflections. The weighted *R*-factor *wR* and goodness of fit *S* are based on *F*^2^, conventional *R*-factors *R* are based on *F*, with *F* set to zero for negative *F*^2^. The threshold expression of *F*^2^ > σ(*F*^2^) is used only for calculating *R*-factors(gt) *etc*. and is not relevant to the choice of reflections for refinement. *R*-factors based on *F*^2^ are statistically about twice as large as those based on *F*, and *R*- factors based on ALL data will be even larger.

### Fractional atomic coordinates and isotropic or equivalent isotropic displacement parameters (Å^2^)

**Table:**

	*x*	*y*	*z*	*U*_iso_*/*U*_eq_	
O1	0.18034 (10)	0.6924 (7)	−0.09151 (11)	0.0931 (9)	
O2	0.08462 (10)	0.6656 (7)	−0.12713 (11)	0.0921 (8)	
N1	0.13189 (12)	0.6134 (7)	−0.08113 (12)	0.0598 (6)	
N2	0.23320 (8)	0.2602 (7)	0.17155 (11)	0.0643 (8)	
H2A	0.2663	0.3390	0.1651	0.077*	
H2B	0.2322	0.1704	0.2138	0.077*	
C1	0.06424 (11)	−0.1027 (8)	0.20919 (14)	0.0592 (8)	
H1A	0.0687	−0.1949	0.2586	0.089*	
H1B	0.0479	−0.2721	0.1723	0.089*	
H1C	0.0377	0.0881	0.2025	0.089*	
C2	0.12500 (10)	0.0091 (7)	0.19996 (12)	0.0489 (7)	
H2C	0.1413	0.1738	0.2388	0.059*	
H2D	0.1515	−0.1852	0.2087	0.059*	
C3	0.12627 (10)	0.1609 (6)	0.12529 (12)	0.0406 (6)	
C4	0.07514 (10)	0.1952 (7)	0.06693 (13)	0.0458 (6)	
H4A	0.0388	0.1174	0.0738	0.055*	
C5	0.07620 (11)	0.3397 (7)	−0.00061 (13)	0.0479 (7)	
H5A	0.0413	0.3608	−0.0389	0.058*	
C6	0.13008 (10)	0.4519 (7)	−0.01007 (12)	0.0445 (6)	
C7	0.18252 (10)	0.4228 (7)	0.04537 (13)	0.0476 (7)	
H7A	0.2185	0.4993	0.0372	0.057*	
C8	0.18102 (10)	0.2780 (7)	0.11353 (12)	0.0442 (6)	

### Atomic displacement parameters (Å^2^)

**Table:**

	*U*^11^	*U*^22^	*U*^33^	*U*^12^	*U*^13^	*U*^23^
O1	0.0812 (15)	0.132 (2)	0.0759 (14)	−0.0154 (16)	0.0390 (12)	0.0189 (15)
O2	0.0889 (15)	0.121 (2)	0.0588 (12)	−0.0001 (15)	0.0045 (11)	0.0277 (14)
N1	0.0728 (16)	0.0621 (16)	0.0473 (12)	−0.0051 (14)	0.0201 (12)	−0.0025 (12)
N2	0.0382 (11)	0.101 (2)	0.0502 (12)	−0.0073 (13)	0.0052 (9)	−0.0046 (14)
C1	0.0702 (18)	0.0533 (18)	0.0597 (16)	−0.0030 (15)	0.0266 (13)	0.0046 (14)
C2	0.0550 (15)	0.0431 (16)	0.0493 (14)	0.0009 (12)	0.0142 (12)	−0.0057 (12)
C3	0.0433 (13)	0.0346 (14)	0.0441 (13)	0.0014 (11)	0.0115 (10)	−0.0080 (11)
C4	0.0402 (13)	0.0443 (16)	0.0529 (14)	−0.0059 (12)	0.0117 (11)	−0.0044 (13)
C5	0.0455 (14)	0.0474 (16)	0.0471 (14)	−0.0007 (13)	0.0044 (11)	−0.0016 (13)
C6	0.0526 (14)	0.0411 (15)	0.0415 (13)	0.0020 (12)	0.0148 (11)	−0.0082 (12)
C7	0.0438 (13)	0.0515 (17)	0.0519 (14)	−0.0063 (13)	0.0202 (11)	−0.0094 (13)
C8	0.0394 (13)	0.0479 (16)	0.0448 (13)	0.0022 (12)	0.0097 (10)	−0.0105 (12)

### Geometric parameters (Å, °)

**Table:**

O1—N1	1.219 (3)	C2—H2C	0.9700
O2—N1	1.218 (3)	C2—H2D	0.9700
N1—C6	1.465 (3)	C3—C4	1.387 (3)
N2—C8	1.395 (3)	C3—C8	1.410 (3)
N2—H2A	0.8600	C4—C5	1.373 (3)
N2—H2B	0.8600	C4—H4A	0.9300
C1—C2	1.517 (3)	C5—C6	1.370 (3)
C1—H1A	0.9600	C5—H5A	0.9300
C1—H1B	0.9600	C6—C7	1.377 (3)
C1—H1C	0.9600	C7—C8	1.387 (3)
C2—C3	1.506 (3)	C7—H7A	0.9300
			
O2—N1—O1	122.8 (2)	C4—C3—C8	117.9 (2)
O2—N1—C6	118.3 (2)	C4—C3—C2	122.5 (2)
O1—N1—C6	118.9 (2)	C8—C3—C2	119.56 (19)
C8—N2—H2A	120.0	C5—C4—C3	122.5 (2)
C8—N2—H2B	120.0	C5—C4—H4A	118.8
H2A—N2—H2B	120.0	C3—C4—H4A	118.8
C2—C1—H1A	109.5	C6—C5—C4	118.2 (2)
C2—C1—H1B	109.5	C6—C5—H5A	120.9
H1A—C1—H1B	109.5	C4—C5—H5A	120.9
C2—C1—H1C	109.5	C5—C6—C7	122.3 (2)
H1A—C1—H1C	109.5	C5—C6—N1	118.9 (2)
H1B—C1—H1C	109.5	C7—C6—N1	118.8 (2)
C3—C2—C1	116.59 (19)	C6—C7—C8	119.2 (2)
C3—C2—H2C	108.1	C6—C7—H7A	120.4
C1—C2—H2C	108.1	C8—C7—H7A	120.4
C3—C2—H2D	108.1	C7—C8—N2	120.0 (2)
C1—C2—H2D	108.1	C7—C8—C3	120.0 (2)
H2C—C2—H2D	107.3	N2—C8—C3	119.9 (2)
			
C1—C2—C3—C4	−0.7 (4)	O1—N1—C6—C7	5.9 (4)
C1—C2—C3—C8	178.4 (2)	C5—C6—C7—C8	−0.6 (4)
C8—C3—C4—C5	−0.5 (4)	N1—C6—C7—C8	178.4 (2)
C2—C3—C4—C5	178.7 (3)	C6—C7—C8—N2	−177.1 (2)
C3—C4—C5—C6	0.3 (4)	C6—C7—C8—C3	0.4 (4)
C4—C5—C6—C7	0.3 (4)	C4—C3—C8—C7	0.1 (3)
C4—C5—C6—N1	−178.7 (2)	C2—C3—C8—C7	−179.0 (2)
O2—N1—C6—C5	5.6 (4)	C4—C3—C8—N2	177.6 (2)
O1—N1—C6—C5	−175.1 (3)	C2—C3—C8—N2	−1.5 (4)
O2—N1—C6—C7	−173.4 (2)		

### Hydrogen-bond geometry (Å, °)

**Table:**

*D*—H···*A*	*D*—H	H···*A*	*D*···*A*	*D*—H···*A*
N2—H2B···N2^i^	0.86	2.62	3.423 (3)	156
C7—H7A···O1^ii^	0.93	2.60	3.417 (4)	147

Symmetry codes: (i) −*x*+1/2, *y*−1/2, −*z*+1/2; (ii) −*x*+1/2, −*y*+3/2, −*z*.
